# Perforating Typhoid Ulcer—Peritonitis—Operation—Recovery

**Published:** 1895-01

**Authors:** Robert Abbe

**Affiliations:** New York


### PERFORATING TYPHOID ULCER—PERITONITIS—OPERATION—RECOVERY.

BY ROBERT ABBE, M. D., NEW YORK.

Late on Saturday afternoon, November 3d, I was asked by Dr. J. H. Bache, of this city, to see with him a young married woman, twenty-one years of age, whom he had been attending with typhoid fever for three weeks. Every characteristic symptom had been present; rather free diarrhœa, evening temperature ranging about 102° F., and delirium every night during the second week. On Monday, October 29th, the patient seemed to be beginning convalescence, all the symptoms abating. Wednesday night she was seized with great pain, as if something had suddenly given way in her abdomen below her navel. She vomited, and was much collapsed. Her temperature rose promptly to 102° F., with great pain and tympanites.

Dr. Bache at once suspected perforation, kept the patient absolutely quiet, and treated her by poultices over the abdomen, and enough morphine to allay her great pain. The temperature fell somewhat, but for two days varied from 102° to 102½° F. Her vomiting was less, but her tympanites continued. At the end of the second day her condition became suddenly worse; the pulse became weaker; the abdomen greatly distended, with skin stretched and shining.

As she had survived two and a half days, Dr. Bache thought, despite her low state, surgery might help her. On examination I found her with mind clear, but heart and respiration oppressed by her distended state; tongue dry and moderately coated; pulse, 140; temperature, 104° F. The distended abdomen was dull in the hypogastrium half-way to the navel, and the dullness extended along the right Poupart ligament. I confirmed the diagnosis and advised the earliest possible operation.

The patient was in too low a state to be removed to a hospital, so the most complete arrangements possible were made to do a thorough operation at the house. In this I was assisted by Drs. A. L. Fisk and Nelson H. Henry, of Trinity Hospital.

A median incision below the navel exposed distended coils of deeply congested and greatly inflamed intestine, smeared with sticky lymph. The pelvis and lower abdomen were filled with a collection of foul, purulent, and fetid intestinal extravasation. This was feebly confined by matted coils of intestines, loosely glued together, that broke apart on being touched, but which, being recognized, enabled me to introduce clean laparotomy sponges under the upper abdominal wall, where a few coils were seen which showed more recent inflammation. Two pints of foul, purulent fluid and thick lymph were cleaned out, and the abdomen irrigated with warm and weak sublimate solution, 1 to 20,000, followed by plain warm-water irrigation.

Search now revealed the cause. The lower part of the ileum showed many thick oval patches in its wall—their long axes parallel to that of the gut, and easily identified by touch as the gut was passed through the fingers, and shown also by increased subperitoneal hyperæmia. One such inflamed Peyer's patch showed a gangrenous perforation a quarter of an inch in diameter, from which intestinal contents were seen to pump out. This was promptly closed by interrupted silk sutures, over which two layers of Halsted mattress stitches were placed, these being found to be the only suture that would hold in the tender and inflamed intestinal coat. A large abdominal tamponade of iodoform gauze was placed within the abdomen and pelvis, and no attempt made to close the wound. A hot black coffee and whisky enema assisted greatly in preventing shock; the patient was put back to bed in three-quarters of an hour from the beginning of etherization.

She passed a good night and had a stronger pulse next morning. Pulse, 132; temperature 102½° F.; tympanites less. At the end of forty-eight hours after operation, as she was in good condition, except for tympanites, I removed the tamponade, reapplied a loose one, and gave five grains of calomel. This produced numerous loose movements and she felt much better. Temperature, 101½° F.; pulse, 120. A little fluid fæces leaked from the wound after the calomel action, showing that the perforation had slightly opened. This continued for two weeks, when it ceased, and the abdominal wound pursued the usual course, closing in rapidly by granulations, and has left a narrow and firm scar.

After the third day her appetite improved and a rapid convalescence ensued.

REMARKS.—The recording of this one case is not an occasion for a long paper on the subject, which would be superfluous in view of the three excellent ones by Mears,[1] Fitz,[2] and Van Hook,[3] written in 1888 and 1891. But it is an opportunity to express a surgical view of the subject more mature than would have been possible a few years since. For, while but few cases of this emergency have as yet been operated on, corresponding conditions of perforating gastric, duodenal, and appendical ulcers have been relatively common, in the hands of the general surgeon. Excepting for the exhausted condition of the patient during typhoid fever, there is practically no difference, as far as I can discern, between the conduct of perforations in various parts of the alimentary tract. The same sudden onset of symptoms is witnessed, acute tearing pain, shock, vomiting followed by tympany and peritonitis. The same sequel pertains as to the peritoneal exudation. In one case, shock, or non-reparative inflammation will succeed and be fatal before operation, or in spite of operation; in another, a fine quality of plastic lymph will repair the damaged part, or hold the extravasation in check until the surgeon can operate. No doubts exist in my own mind that recovery without operation can follow perforation anywhere—and in typhoid also. But this is certainly infinitely rarer here than in other diseases, because the parts are centrally located where the explosion occurs and a natural outlet through the abdominal wall does not easily take place. The case narrated later, where a typhoid ulcer perforated and a resulting abscess was opened in the perineum, is consistent with much of nature's good work.

Fitz says: "The similarity of the symptoms of typhoid perforations of the bowel and those of the appendix, is striking. Cases of perforating appendix have repeatedly been regarded as typhoid fever, and, as a rule, the symptoms of typhoid which suggest perforation of the bowels, are those which, in the absence of typhoid, would be regarded as diagnostic of

---

1 J. Ewing Mears: Transactions of the American Surgical Association, 1888.

2 Fitz: Transactions of the Association of American Physicians, 1891, vol. vi., p. 200.

3 Philadelphia Medical News, 1891, vol. ix., p. 591. Chicago Medical Record, 1891, vol. ii., pp. 229-270.

appendicitis. The symptoms are not merely similar, they are actually identical. Even to the usual localization of the consequent peritonitis to the right iliac fossa."

This lucid statement by Fitz must appeal to every observer of appendicitis cases, as true to the letter. Why one class of cases should be left to die, while we operate on all appendicitis cases when perforation can be recognized, does not appear. The typhoid statistics are improving, and as it was in the other, we may here also soon record as much gain in the near future.

Van Hook operated on a desperate case at two o'clock in the afternoon, where perforation had taken place at five a. m. The extravasation was wide of the ulcer. The patient was washed out with plain boiled water, sewed up tight, rescued from collapse, and saved.

Now, we have not yet learned enough about the surgical procedure to do exactly right in all cases, and it gives me some satisfaction to state in a few words what I regard of growing importance in the work. The technical points are known to every surgeon, but very essential do I consider it, that the surgeon should never be so hasty in getting at his work that he enters upon it handicapped by poor assistance, poor light, poor arrangements for irrigation and sponging, or inadequate plans for restoration from shock.

I regard the rescue of these cases from collapse as most important. I always give a restorative enema at the end of operation (often on the table), composed of a cup of hot black coffee and one or two ounces of whisky, followed later by hypodermics of strychnine and digitalis, abundant heat about the body, and elevation of the foot of the bed for twelve hours. Very warm saline infusion into the vein, of at least one pint, must always be tried as a valuable resort.

To quote from Dr. Van Hook's article, the statistics up to October, 1891, of operation for typhoid perforation were:

"1884. Mikulicz, four cases, one recovery, though, unfortunately, the diagnosis is doubtful.

1885. Lucke, one case, resection, death.

1886. Escher, one case, recovery, but the case is regarded by Louis as one of appendicitis.

1886. Greig Smith, one case, doubtful diagnosis, death.

1886. Bartlett, one case, death.

1887. Bontecou, one case, death.
1887. Morton, one case, death.
1889. Bontecou, one case, death.
1889. Senn, volvulus and perforation, one case, death.
1889. Hahn, two cases, death.
1890. Kimura, one case, death.
1890. Taylor, one case, recovered.
1890. Van Hook, three cases, two dead, one recovered.

Making in all, nineteen laparotomies and four recoveries.

"Mikulicz's case was said by the author to be of doubtful origin. That of Escher was probably a perforative appendicitis. Taylor's case should not be counted, as it was operated on ten days after onset of symptoms, and is not demonstrated to have been typhoid perforation.

"If we include all the doubtful cases, the present recovery (Van Hook's case), as appears from the literature at command, is the nineteenth case and fourth recovery. If we include only closely diagnosticated cases, it is the twelfth case and first recovery."

In 1894, Cayley[1] and H. Allingham[2] each reported a fatal case after early operation, stitching the gut to the abdominal wound.

In *The Lancet*, 1894, vol. i., p. 1615, is recorded a case of supposed perforation with collapse and recovery, in a female, which I am strongly inclined to regard as one of high intestinal hemorrhage in the fourth week, indicated by a drop in temperature to 95°, rapid, thready pulse, cold extremities and extreme pallor, and fullness of the umbilical region and tenderness in the right hypochondrium. No pain existed and the patient made a slow recovery with a very thin pulse. No operation was performed.

In August, 1893, Dr. Newton, of Chicago,[3] treated a case of supposed perforation by morphine; as the parents objected to the operation. The case seems undoubted, and the boy recovered, an abscess of the pelvis being opened a fortnight later in the perineum.

Netschagaw, of St. Petersburg, records a case this year of resection of a portion of perforated bowel in typhoid, with recovery.

---

1 British Medical Journal, 1894, vol. i., p. 578.
2 Ibid.
3 Chicago Medical Recorder, 1893, vol. v., p. 409.

Since Van Hook's successful case in 1891, there have been reported the following operations:

1894. Cayley and Bland Sutton, one case, fatal.
1894. H. Allingham, one case, fatal.
1894. Netschagaw, Medical News, December 1, 1894, p. 609, one case, recovery.
1894. Abbe, one case, recovery.
1894. Alexandroff,[1] one case, death.

If we accept all the nineteen laparotomies for so-called typhoid perforations with four recoveries, collected by Van Hook, and add the five new ones, we have 24 cases and 6 recoveries.

If we throw out doubtful cases—which I agree with Van Hook should be done—and accept only 12 of the 19 with 1 recovery—to which the above five are added—then the correct statistics revised up to date, stand, 17 cases with 3 recoveries. —*Medical Record.*

---

1 Clinical and Therapeutical Journal, vol. ii., p. 735. Paris, 1894.

